# Current therapeutic strategies in Parkinson’s disease: Future perspectives

**DOI:** 10.1016/j.mocell.2025.100274

**Published:** 2025-09-04

**Authors:** Tae Young Kim, Byoung Dae Lee

**Affiliations:** 1 Department of Neuroscience, Kyung Hee University, Seoul, South Korea; 2 Department of Physiology, Kyung Hee University School of Medicine, Seoul, South Korea

**Keywords:** Cell replacement therapy, Dopaminergic neurons, Neurodegeneration, Parkinson's disease, Pluripotent stem cell

## Abstract

Parkinson’s disease (PD) is a progressive neurodegenerative disorder characterized by the loss of dopaminergic neurons and the accumulation of misfolded α-synuclein. Current treatments, including dopaminergic medications and deep brain stimulation, provide symptomatic relief but do not halt disease progression. Recent advances in molecular research have enabled the development of disease-modifying strategies targeting key pathogenic mechanisms, such as α-synuclein aggregation, mitochondrial dysfunction, and genetic mutations, including *LRRK2* and *GBA1*. In parallel, pluripotent stem cell-derived dopaminergic neurons have emerged as a scalable and ethically viable source for cell replacement therapy. Early-phase clinical trials have demonstrated the safety and functional integration of these grafts. Ongoing research is now focused on enhancing graft purity, immune compatibility, and anatomical precision, including homotopic transplantation and circuit-level reconstruction. Together, these emerging strategies offer the potential to shift PD treatment paradigms by combining symptomatic control with long-term neural restoration. This review summarizes current therapeutic approaches and highlights recent advances in disease-modifying and regenerative interventions for PD.

## INTRODUCTION

Parkinson’s disease (PD) is a prevalent and progressive neurodegenerative disorder that primarily affects the motor system but also leads to a broad range of nonmotor symptoms, including cognitive decline, mood disturbances, and autonomic dysfunction (Schapira et al., 2017). The disease is pathologically defined by the loss of dopaminergic (DA) neurons in the substantia nigra pars compacta (SNpc) and the accumulation of intracellular protein aggregates known as Lewy bodies. The pathogenesis of PD is multifactorial, involving genetic mutations, environmental exposures, mitochondrial dysfunction, oxidative stress, neuroinflammation, and impaired protein homeostasis. Several genetic risk factors, including mutations in the *leucine-rich repeat kinse 2* (*LRRK2)* and *glucosylceramidase* (*GBA1)* genes, have been linked to both familial and sporadic PD, underscoring the complexity of the underlying disease mechanisms (Di Maio et al., 2018, Eblan et al., 2005, Sidransky et al., 2009).

Current therapies for PD are largely symptomatic and focus on restoring DA signaling through pharmacological agents or neurosurgical interventions. While these approaches provide substantial relief of motor symptoms, they do not modify the disease course or prevent neurodegeneration. Furthermore, long-term treatment is often complicated by adverse effects and motor fluctuations (Rampello et al., 1996, Thanvi and Lo, 2004), highlighting the urgent need for disease-modifying and restorative strategies. Recent advances in molecular biology, immunology, and regenerative medicine have led to the development of promising experimental therapies aimed at targeting core pathological mechanisms. These include immunotherapies against α-synuclein (α-syn), gene therapies to correct or mitigate genetic risk factors, and cell replacement strategies using fetal or pluripotent stem cell (PSC)-derived DA neurons. Together, these emerging modalities offer hope for not only improving symptom management but also altering the trajectory of PD progression. This review summarizes the current state of clinical therapeutics for PD and highlights recent progress in disease-modifying interventions and stem cell–based regenerative therapies.

## CURRENT CLINICAL THERAPEUTICS

Current therapeutic approaches are broadly categorized into DA therapies, nondopaminergic (ND) symptomatic treatments, and surgical interventions. Table 1 provides an overview of these therapeutic modalities, summarizing their clinical applications along with associated advantages and limitations.

**Table 1:** Summary of current clinical applications

	Treatment	Description	Advantages	Limitations
Dopaminergic therapies	Levodopa	Dopamine precursor, typically coadministered with a peripheral dopa-decarboxylase inhibitor (eg, carbidopa or benserazide) to prevent peripheral metabolism	Rapid and reliable relief of motor symptoms	Long-term use is associated with motor complications, such as wearing-off phenomena and dyskinesias
	Dopamine agonists (pramipexole, ropinirole, and rotigotine)	Often used in early-stage PD or as adjuncts to levodopa to reduce motor fluctuations	Longer half-life than levodopa, reduced risk of early motor complications	Associated with impulse control disorders, hallucinations, and sleep disturbances
	MAO-B inhibitors (selegiline, rasagiline)	Inhibit dopamine breakdown and prolong its effect	Can be used as monotherapy in early PD, delays the initiation of levodopa	Modest efficacy, potential for drug interactions
	COMT inhibitors (entacapone, opicapone)	Reduce peripheral degradation of levodopa, extending its half-life and minimizing “off” periods	Useful adjunct to levodopa for patients with motor fluctuations	May cause diarrhea, urine discoloration, and hepatotoxicity (especially with tolcapone)
Nondopaminergic symptomatic therapies	Anticholinergics (trihexyphenidyl, benztropine)	Primarily used for tremor control in younger patients	Effective for tremor	Poor tolerability in older adults due to cognitive side effects
	Amantadine	Originally developed as an antiviral, exerts weak dopaminergic and NMDA antagonist effects	Reduces dyskinesia; has modest antiparkinsonian efficacy	Can cause hallucinations, peripheral edema, and livedo reticularis
	Adenosine A2A receptor antagonists	Approved in some countries as adjunctive therapy to reduce “off” time	Add-on benefit to levodopa, may improve motor function	Limited efficacy, high cost, insomnia, and hallucinations can occur
Surgical interventions	Deep brain stimulation	High-frequency stimulation of the subthalamic nucleus or globus pallidus interna	Significantly improves motor symptoms, reduces medication needs, and enhances quality of life	Surgical complications, side effects (cognitive impairments, memory problems, and mood changes)
	Lesional therapies	Radiofrequency ablation and focused ultrasound thalamotomy or pallidotomy are used in select patients, particularly those with tremor-dominant PD who are ineligible for DBS	Noninvasive options (eg, ultrasound) are emerging, effective in reducing tremor	Irreversible, usually limited to unilateral treatment

COMT, catechol-O-methyltransferase; DBS, deep brain stimulation; MAO-B, monoamine oxidase-B; NMDA, N-methyl-D-aspartate; PD, Parkinson’s disease.

### Dopaminergic and Nondopaminergic Therapies

DA therapies remain the cornerstone of PD treatment. Levodopa (L-dopa), the metabolic precursor of dopamine, is the most effective agent for alleviating bradykinesia and other cardinal motor symptoms. Unlike dopamine, L-dopa can cross the blood-brain barrier (BBB) and is subsequently converted to dopamine within the brain. To enhance central bioavailability and reduce peripheral side effects, it is coadministered with dopa-decarboxylase inhibitors, which block peripheral conversion of L-dopa to dopamine. Although the results remain somewhat controversial, emerging evidence suggests that L-dopa may exhibit disease-modifying properties or confer sustained benefits even after discontinuation (Verschuur et al., 2015). However, long-term use is associated with complications such as nausea, somnolence, and, particularly, in older patients, cognitive disturbances including hallucinations and psychosis (Klietz et al., 2019). Other DA agents include dopamine agonists, monoamine oxidase-B (MAO-B) inhibitors, and catechol-O-methyltransferase (COMT) inhibitors. MAO-B inhibitors enhance DA tone by inhibiting dopamine breakdown and are used either as monotherapy in early PD or in combination with L-dopa to reduce motor fluctuations. Specifically, they help minimize “off” periods (times of reduced motor control) and extend “on” time (periods of good symptom control) (Tan et al., 2022). COMT inhibitors prolong the effect of L-dopa by inhibiting its peripheral degradation, thereby helping to manage “wearing-off” episodes, in which the efficacy of L-dopa diminishes before the next dose (Jenner et al., 2021).

ND symptomatic treatments have gained attention due to the involvement of multiple neurotransmitter systems in PD pathophysiology. Although they generally confer less pronounced motor improvement than L-dopa, ND agents contribute meaningfully to the management of both motor and nonmotor symptoms (Isaacson and Jenner, 2025). Anticholinergics are primarily used to control tremor, particularly in younger patients, but their application is limited in older patients due to the heightened risk of cognitive side effects. Amantadine, an N-methyl-D-aspartate (NMDA) receptor antagonist, is employed to reduce L-dopa-induced dyskinesia and may also provide modest improvements in rigidity and tremor (Wang et al., 2022). Adenosine A2A receptor antagonists represent an emerging therapeutic class, shown to reduce “off” time without exacerbating dyskinesias (Jenner, 2014). Additionally, ND therapies are utilized to manage target-specific nonmotor symptoms such as cognitive dysfunction, orthostatic hypotension, and sialorrhea, although therapeutic efficacy in these domains remains variable and often patient-dependent.

### Surgical Interventions

**Fig. 1:**
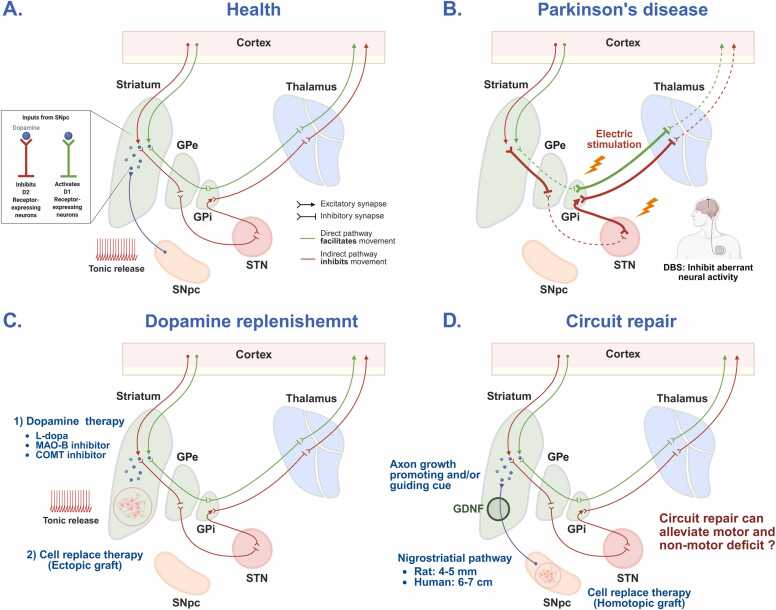
Dopaminergic regulation of the basal ganglia motor circuit and therapeutic strategies for Parkinson’s disease (PD). (A) *Basal ganglia circuitry under normal conditions*. The basal ganglia control movement through direct and indirect pathways originating from the striatum. Dopamine from the substantia nigra pars compacta (SNpc) modulates these pathways by activating D1 receptors (direct pathway, promoting movement) and D2 receptors (indirect pathway, suppressing movement). The globus pallidus internus (GPi) exerts tonic inhibition on the thalamus, which regulates motor cortex activity. Dopaminergic modulation ensures proper movement initiation and suppression. (B) *Circuit dysfunction in PD*. Loss of SNpc neurons reduces striatal dopamine, weakening the direct pathway and overactivating the indirect pathway. This enhances GPi-mediated thalamic inhibition, resulting in motor symptoms such as bradykinesia and rigidity. Deep brain stimulation (DBS) of the STN or GPi helps restore balance by suppressing pathological overactivity. (C) *Dopamine restoration therapies*. L-Dopa and striatal cell transplantation aim to restore dopamine levels and re-establish functional output of basal ganglia circuits. (D) *Intranigral grafting for circuit reconstruction*. Transplanting dopaminergic neurons into the SN may enable reformation of the nigrostriatal pathway, restoring more physiological connectivity. While effective in rodents, the longer axonal distances in humans present a challenge. Adjunctive approaches using neurotrophic factors, axon guidance molecules, or biomaterials may support long-distance axonal growth and integration in clinical settings. GDNF, glial cell line-derived neurotrophic factor; GPe, globus pallidus externus. Created in BioRender (https://BioRender.com/rhaheq1).

Surgical interventions, particularly deep brain stimulation (DBS), are typically considered for patients with advanced PD who experience disabling motor fluctuations or medication-refractory tremor. The subthalamic nucleus (STN) emerged as a principal DBS target following a landmark study by Bergman et al. (1990), in which STN lesions in 1-methyl-4-phenyl-1.2.3.6-tetrahydropyridine (MPTP)-treated primates resulted in marked improvement in PD-like motor symptoms, including tremor, rigidity, and bradykinesia. Subsequent research employing both surgical ablation and high-frequency stimulation further validated the STN as a therapeutic target (Aziz et al., 1991, Benazzouz et al., 1993). DBS involves stereotactic implantation of electrodes into deep brain structures such as the STN or globus pallidus internus. Continuous high-frequency electrical stimulation modulates aberrant neuronal activity within the basal ganglia circuitry ( Fig. 1A and B) (Rajamani et al., 2024). Clinically, DBS has been shown to significantly improve motor symptoms, reduce “off” periods, and permit reduction in DA medication dosage, thereby decreasing medication-related complications (Bari et al., 2018, Deuschl et al., 2006).

## EMERGING DISEASE-MODIFYING AND EXPERIMENTAL THERAPIES

Recent advances in molecular neuroscience and translational research have catalyzed the development of disease-modifying strategies aimed at targeting key pathogenic mechanisms in PD. These include abnormal aggregation of α-syn, mitochondrial dysfunction, chronic neuroinflammation, and genetic mutations such as *LRRK2* and *GBA1*. Several therapeutic candidates are currently under investigation in preclinical and clinical settings, with the goal of slowing or halting disease progression rather than merely alleviating symptoms. Table 2 summarizes the current landscape and development status of prominent clinical trials evaluating disease-modifying agents in PD.

**Table 2:** Summary of current status of prominent clinical trials

Category	Agent	Description	Clinical trial (readout, endpoint)
PD gene	Buntanetap	Suppresses the translation of the mRNAs of APP, tau, α-syn, and other neurotoxic aggregating proteins	Phase III (2024); improvements in motor and nonmotor activities, as well as cognitive functions, safety/tolerability
	Minzasolmin	α-Syn misfolding inhibitor	Phase IIa (2024); failed. Does not meet primary and secondary outcome, progression in MDS-UPDRS part I-III sum score over 12-18 months, safety, and DAT-SPECT
	Prasinezumab	Aggregated/fibrillar (pathological selective), C-terminal (aa 118-126), seed neutralization, propagation block, and Fc-mediated clearance	Phase II (2024); slow motor progression in rapidly processing early-stage PD, change from baseline in MDS-UPDRS part III (OFF), safety, and DAT-SPECT
	Cinpanemab (BIIB054)	Aggregated, N-terminal (aa 1-10), and seed neutralization/propagation block	Phase II (2024) negative, change in MDS-UPDRS part III (OFF) in 72 weeks, safety, PK/PD, and imaging
	MEDI1341 (Astrazeneca/Takeda)	Pathological/aggregated-selective, conformational/C-terminal biased, seed sequestration, and propagation attenuation	Phase I (2022) completed, safety/tolerability, PK/PD, immunogenicity, and CSF target engagement
	ABBV-0805 (BAN0805, BioArctic)	Oligomer/protofibril-selective, conformational, seed neutralization, and strong microglial uptake of soluble aggregates	Phase I (2020) initiated, safety/tolerability, PK/PD, immunogenicity, and CSF biomarkers
	BIIB122	LRRK2 kinase inhibitor	Phase I (2023); safety/tolerability, Phase IIb (start in 2024); underway, PD patients with or without LRRK2 variant, MDS-UPDRS-based efficacy endpoint, and PK/PD
	NEU-723	LRRK2 kinase inhibitor	Phase I, safety/tolerability, PK/PD, and biomarker readout
	ARV-102	LRRK2 inhibition, PROTAC	Phase I (2025); safety/tolerability, LRRK2 reduction, PK/PD, and CNS penetration
	BIA 28-6156 (LTI-291)	GCase activator, oral allosteric activator	Phase II (2023), meaningful motor progression, MDS-UPDRS change, Hoehn-Yahr, PDQ-39, CGI/PGI, safety, and PK/PD
	GT-02287	GCase modulator	Phase Ib (2024), safety/tolerability
Drug reposition	Ambroxol	Cough suppressant, enhance GBA (GCase) activity, and remove waste proteins	Phase II (2020), safety/tolerability Phase III (start in 2025); underway, rate of change in MDS-UPDRS part III (OFF) to 104 weeks, MoCA, PDQ-39, and CGI/PGI
	Exenatide	GLP-1 receptor activator, improve memory, and reduce inflammation and α-syn	Phase III (2024); failed, does not meet primary and secondary outcomes, difference in MDS-UPDRS Part III (OFF) at 96 weeks, and MoCA
	Liraglutide	GLP-1 receptor activator	Phase II (2022); improve nonmotor symptoms, overall mobility, nonmotor and activities-of-daily-living outcomes, MDS-UPDRS, PDQ-39, and safety
	Lixisenatide	GLP-1 receptor activator, less motor disability progression	Phase II (2024); slow progression of movement symptom, moving to phase III, change in MDS-UPDRS part III (ON) at 12 months, LEDD, and safety
Inflammation	VTX3232	NLRP3 (a part of a molecular complex, inflammasome) inhibitor	Phase I; safety/tolerability, Phase IIa (start in 2024); underway, early PD patients
	Dapansutrile	NLRP3 inhibitor, reduce inflammation	Phase II (start in 2024); underway, safety/tolerability, MDS-UPDRS, and inflammatory biomarkers
Gene therapy	AAV2-GDNF	GDNF, a protein supports and nurtures brain cells	Phase II (2022); safety/tolerability up to 5 years, underway, MDS-UPDRS, and imaging
	AAV8-GBA1	Administer the normal GBA gene in PD patients with at least on GBA1 variant loci	Phase I/IIa (start in 2020); underway, safety/tolerability, CSF GCase activity, imaging, and LEDD
	AAV-GAD	Glutamic acid decarboxylase (GAD), increase the production of GABA introduced into STN	Phase I/II (2024); safety/tolerability, improve movement, and PET/metabolic imaging
Mitochondria	UDCA	Ursodeoxycholic acid (UDCA), bile acid currently prescribed to treat liver disease, improve mitochondria integrity and function	Phase II (2023); safety/tolerability, improve cell energy production, and possible improvement in gait
	MTX325	Targets USP30, an enzyme that inhibits mitophagy, improves mitochondrial quality and function by enhancing mitophagy	Phase I (2024): safety/tolerability, PK/PD, and mitochondria biomarker

APP, amyloid β precursor protein; MDS-UPDRS, movement disorders society-unified Parkinson's disease rating scale; DAT-SPECT, dopamine transporter-single photon emission computed tomography; CSF, cerebrospinal fluid; LRRK2, leucine-rich repeat kinase 2; PROTAC, proteolysis targeting chimera; CNS, central nervous system; GCase, glucocerebrosidase; PDQ-39, Parkinson's disease questionnaire; CGI/PGI, clinical global impressions-global improvement/patient global impression of improvement; GBA, glucosylceramidase; MoCA, montreal cognitive assessment; GLP-1, glucagon-like peptide-1; LEDD, l-dopa equivalent daily dose; NLRP3, NLR family pyrin domain containing 3; AAV, adeno-associated virus; GDNF, glial cell line-derived neurotrophic factor; PD, Parkinson's disease; GABA, γ-aminobutyric acid; STN, subthalamic nucleus ; USP30, ubiquitin specific peptidase 30.

### Targeting α-Synuclein Aggregation

The accumulation of misfolded α-syn into Lewy bodies and Lewy neurites represents a pathological hallmark of PD and related α-synucleinopathies, including dementia with Lewy bodies, multiple-system atrophy, and rapid eye movement (REM) sleep behavior disorder (Calabresi et al., 2023). While compelling evidence implicates α-syn aggregation to PD progression, its precise pathological role and the most effective therapeutic targeting strategies remain incompletely understood. Current investigational approaches fall into two categories: immunotherapies and small-molecule inhibitors. In contrast to small-molecule approaches that primarily act inside neurons, anti–α-syn monoclonal antibodies act predominantly in the extracellular space, where pathogenic α-syn assemblies circulate and mediate cell-to-cell transmission. By binding and neutralizing extracellular seeds, antibodies can (1) limit interactions with neuronal uptake pathways and thereby reduce internalization and seeding; (2) facilitate Fc-dependent microglial clearance; and (3) lower the interstitial/CSF seed burden that drives network-level spread. Because α-synucleinopathy is thought to progress along Braak-like regional staging, targeting the extracellular transmissible pool provides a plausible disease-modifying strategy to slow transneuronal spread and thereby attenuate stagewise progression. These extracellular mechanisms are complementary to intracellular small-molecule (and oligonucleotide) modalities and are well-suited for combination frameworks, including peri- and post-transplant use alongside cell replacement therapy (CRT). Immunotherapy strategies include active immunization, which stimulates the host immune system to produce antibodies against α-syn, and passive immunization, which involves the administration of exogenous monoclonal antibodies targeting pathogenic α-syn conformers. Clinical-stage monoclonal antibodies can be organized by (1) targeted α-syn species—“total/monomeric,” oligomer-/protofibril-selective, or fibril-/aggregated-selective—and (2) epitope—N-terminal vs C-terminal or conformational (Table 2). Among these, Prasinezumab, developed by Roche, represents one of the most advanced clinical candidates. Although the Phase II PASADENA trial did not meet its primary endpoint, it indicated a potential for delaying motor progression in early-stage, treatment-naive patients or those receiving MAO-B inhibitors, leading to study extension through 2026 (Pagano et al., 2022, Pagano et al., 2024). In the subsequent Phase IIb PADOVA trial, Prasinezumab showed a delay in the time to confirmed motor progression, particularly among patients concurrently treated with L-dopa, suggesting a possible therapeutic window for enhanced efficacy (Nikolcheva et al., 2025). Beyond antibodies, several small-molecule strategies are in active investigation. First, aggregation/misfolding modulators include minzasolmin (UCB0599/NPT200-11), an oral inhibitor of α-syn misfolding (Table 2) (Smit et al., 2022). Anle138b, an orally available oligomer modulator, has completed Phase I and is advancing in patient studies (Levin et al., 2022). Second, propagation-focused approaches aim to reduce membrane-bound α-syn or block its spread; ENT-01 (squalamine phosphate) improved gastrointestinal motility and showed neurological signals in a randomized Phase IIb PD constipation trial (Camilleri et al., 2022). Third, lysosomal enhancement with ambroxol (a GCase chaperone) demonstrates target engagement in PD and is under evaluation in genotype-informed trials (Colucci et al., 2023). Finally, innate-immune/propagation blockade with small-molecule TLR2/TLR9 antagonists (eg, NPT1220-312) has shown potent inhibition of proinflammatory signaling in human microglia and blood ex vivo, supporting further translational study (Habas et al., 2022). Collectively, these modalities broaden the α-syn-targeting landscape beyond antibodies and are well-suited to combination frameworks alongside CRT.

### Targeting Genetic Drivers—LRRK2 and GBA

Gain-of-function mutations in *LRRK2*, particularly the G2019S variant, result in kinase hyperactivation that contributes to mitochondrial dysfunction, autophagy-lysosomal impairment, and exacerbation of α-syn pathology (Taymans et al., 2023). To counteract these effects, several orally bioavailable LRRK2 kinase inhibitors have been developed. Among them, DNL201 and DNL151 (Denali Therapeutics), later acquired by Biogen, demonstrated favorable safety and pharmacodynamic profiles in Phase I clinical trial (Jennings et al., 2023). The most advanced compound, BIIB122/DNL151, a brain-penetrant LRRK2 inhibitor, is currently being evaluated in a global Phase IIb clinical trial (LUMA study) enrolling early-stage PD patients, irrespective of their *LRRK2* mutation status. Additional industry efforts include Neuron23's NEU-723 (Phase I first-in-human initiated; precision-medicine stratification under development). Tool/legacy compounds (eg, MLi-2, PFE-360) have informed dose selection and class biology, including attention to on-target effects in peripheral tissues. However, concerns remain regarding the long-term safety of systemic LRRK2 inhibition, particularly due to its expression in peripheral organs such as the lung and kidney.

Mutations in *GBA1*, which encodes the lysosomal enzyme glucocerebrosidase (GCase), lead to reduced enzymatic activity, impaired lysosomal degradation, and subsequent accumulation of α-syn, thereby promoting neurodegeneration (Zhang et al., 2024). Therapeutic efforts to restore GCase function encompass both small molecules and gene therapy. Ambroxol, a pharmacological chaperone, enhances GCase folding and trafficking, thereby enhancing lysosomal function (Kopytova et al., 2021). It has demonstrated CNS penetration and positive effects on GCase activity and α-syn clearance in early clinical studies. The Phase II study confirmed its safety and target engagement, and a Phase III trial is currently ongoing (Colucci et al., 2023). BIA 28-6156 (formerly LTI-291) is an oral allosteric GCase activator now in Phase II after Phase Ib studies showed CNS exposure and pharmacodynamic effects. Concurrently, gene therapy approaches, such as PR001 from Prevail therapeutics, utilize adeno-associated virus vectors to deliver functional GBA1 directly to the CNS, with early-phase clinical trials underway.

### GLP-1 Receptor Agonists and Glial Inflammation

GLP-1 receptor agonists (GLP-1RAs), originally developed for metabolic disease, exhibit neuroprotective and anti-inflammatory actions in preclinical PD models by engaging GLP-1 receptors on microglia and astrocytes, dampening NF-κB/NLRP3 signaling, shifting microglia toward pro-resolving states, and supporting mitochondrial and lysosomal function—mechanisms that align with a disease-modifying potential in PD (Roy et al., 2025). Clinically, the Phase II LIXIPARK trial reported less progression of motor disability at 12 months with lixisenatide vs placebo (with expected gastrointestinal adverse events), supporting continued evaluation in larger, longer studies (Meissner et al., 2024). In contrast, the Phase 3 Exenatide-PD3 trial found no evidence that weekly exenatide slowed PD progression vs placebo over 96 weeks, underscoring possible drug- and population-specific effects within the class and the need for biomarker-anchored designs (Vijiaratnam et al., 2025). Additional GLP-1RA programs—including semaglutide (NCT03659682) and liraglutide (NCT02953665)—are ongoing and will help clarify class heterogeneity, optimal patient selection, and mechanistic endpoints linked to neuroinflammation. Taken together, GLP-1RAs represent a complementary, inflammation-targeted modality that can be considered for combination frameworks (eg, alongside α-syn-directed agents or CRT), provided future trials confirm durable clinical benefit and delineate responders using immune and neuronal biomarkers.

## CELL REPLACEMENT THERAPY

The concept of CRT for PD originated from pivotal studies in 1979 demonstrating that intracerebral grafting of fetal mesencephalic tissue—rich in DA neuroblasts—could alleviate motor deficits in the 6-OHDA-lesioned rat model of PD (Bjorklund and Stenevi, 1979, Perlow et al., 1979). These foundational experiments established the feasibility of restoring DA input to the striatum via cellular transplantation. Since then, numerous cell sources have been investigated as potential therapeutic candidates for CRT, including fetal ventral mesencephalic tissue, autologous and allogenic stem cells, and more recently, human pluripotent stem cell (hPSC)-derived DA neurons. These efforts aim to replace the degenerating nigrostriatal DA neurons and restore functional motor circuitry in PD (Fig. 1A and C).

### Early Foundation—Fetal Tissue Transplantation

Open-label clinical trials, most notably those conducted by Lindvall and colleagues, demonstrated that human fetal ventral mesencephalic (VM) DA neurons—harvested from embryos at 8-9 weeks of gestation—could survive transplantation into the striatum of PD patients and confer meaningful clinical benefits (Lindvall et al., 1990, Lindvall et al., 1989, Lindvall et al., 1992). These grafts not only synthesized and released dopamine but also led to significant motor symptom improvement, with some patients maintaining therapeutic gains for over two decades (Barker et al., 2015). Postmortem analysis confirmed the long-term survival and integration of the transplanted neurons, including evidence of axonal extension and synaptic connectivity within host brain circuits (Li et al., 2016). Despite these promising findings, fetal tissue transplantation encountered major obstacles that hindered its broader clinical implementation. Ethical controversies surrounding the use of aborted fetal tissue, alongside logistical challenges such as limited donor availability, inconsistent tissue quality and developmental stage, and variable DA cell content, posed substantial barriers to standardization and scalability. Additionally, clinical outcomes were heterogeneous, and subsequent double-blind, placebo-controlled trials produced mixed results, casting uncertainty on the reliability of this approach. Collectively, these challenges underscored the pressing need for ethically acceptable, scalable, and functionally robust sources of human DA neurons to advance CRT for PD.

### Pluripotent Stem Cells—A Scalable and Ethical Alternative

PSC-based strategies, utilizing either embryonic stem cells (ESCs) or induced pluripotent stem cells (iPSCs), offer a scalable and ethically feasible alternative to fetal tissue for CRT in PD. ESCs are derived from the inner cell mass of early-stage embryos (Thomson et al., 1998), whereas iPSCs are generated by reprogramming somatic cells to a pluripotent state (Takahashi et al., 2007). Despite the promise of PSCs, several challenges must be addressed to ensure clinical safety and efficacy. These include the risk of tumorigenicity from residual undifferentiated cells and the presence of contaminating serotonergic neurons, which have been implicated in graft-induced dyskinesia—an adverse effect previously observed in fetal VM transplants. Consequently, producing homogeneous, well-characterized cell populations is critical.

Mesencephalic dopaminergic (mesDA) neurons comprise 3 anatomically and functionally distinct clusters—retrorubral field (A8 group), the SNpc (A9 group), and the VTA (A10 group)—defined by molecular signatures and electrophysiological properties. Among these, A9 neurons are of particular interest due to their selective vulnerability in PD. To generate authentic mesDA neurons from hPSCs, differentiation protocols grounded in developmental biology typically begin with dual SMAD (an acronym from the fusion of *Caenorhabditis elegans Sma* genes and the *Drosophila Mad*, Mothers against decapentaplegic) inhibition to promote neuroectodermal differentiation while suppressing mesodermal and endodermal lineages (Chambers et al., 2009). Subsequent, precisely timed activation of WNT (wingless-related integration site) and Sonic Hedgehog signaling steers cells through a mesencephalic floorplate intermediate toward ventral midbrain identity and A9-like fate (Doi et al., 2014, Kirkeby et al., 2012, Kriks et al., 2011). This strategy yields hPSC-derived mesDA neurons that exhibit robust survival, long-range axonal integration, and functional motor recovery in PD models (Chen et al., 2016, Kikuchi et al., 2017, Kirkeby et al., 2017). Building on these preclinical successes, extensive process development has culminated in Good Manufacturing Practice-compliant, clinically scalable protocols, enabling early-phase human trials and marking a significant translational milestone for stem cell–based regenerative therapies in PD.

### Immune Compatibility and Transplantation Strategies

Although the CNS has long been considered as immune-privileged site due to its limited lymphatic drainage and the protective function of the BBB, recent discoveries have challenged this paradigm. The identification of the glymphatic system (Yang et al., 2013) and CNS lymphatic vessels (Louveau et al., 2015) suggests that immune surveillance in the brain is more dynamic than previously thought. Furthermore, surgical disruption of the BBB during intracerebral transplantation can expose grafted cells to peripheral immune components, increasing the risk of immune rejection. Autologous transplantation using patient-specific iPSCs offers the theoretical advantage of immune compatibility. However, this approach presents several practical challenges, including high production costs, lengthy preparation timelines, and potential reintroduction of disease-associated genetic mutations. In contrast, allogeneic transplantation requires immunosuppressive therapy to prevent host-vs-graft responses. To address the immunogenicity barrier while maintaining feasibility for broad clinical use, several countries have initiated the development of iPSC banks derived from human leukocyte antigen (HLA)-homozygous “super donors” (Wilmut et al., 2015). These donors, often blood type O and homozygous at key HLA loci, offer broad immunological compatibility, enabling the production of off-the-shelf cell products with reduced rejection risk (Parmar et al., 2020). Nevertheless, even partial HLA mismatches may necessitate immunosuppressive regimens to ensure long-term graft survival and functional integration.

### Clinical Trials and Recent Milestones

Recent results from 2 clinical trials (Sawamoto et al., 2025, Tabar et al., 2025) highlight the translational progress of PSC-based CTR therapy for PD. In an investigator-initiated, open-label, single-center phase I/II study at Kyoto University, CORIN-sorted allogeneic iPSC-derived DA progenitors were bilaterally transplanted into the putamen of 7 patients (ages 50-69) at 2 dose tiers (2.1-2.6 × 10^6^ vs 5.3-5.5 × 10^6^ cells per hemisphere). Tacrolimus immunosuppression was tapered at 12 months and stopped at 15 months. The primary endpoint was safety/tolerability; secondary endpoints included MDS-UPDRS III (ON/OFF) and multimodal PET (^18^F-DOPA for dopamine synthesis, ^18^F-FLT for proliferation, and ^18^F-GE180 for microglial activation). Over 24 months, there were no serious adverse events, no tumor-like enlargement on MRI, and one moderate dyskinesia; putaminal ^18^F-DOPA uptake increased by 44.7% in a dose-responsive manner, and mean motor scores improved by 9.5 points (20.4%) OFF and 4.3 points (35.7%) ON among 6 efficacy-evaluable participants (Sawamoto et al., 2025). In parallel, a multisite, open-label Phase I trial of an hESC-derived product (bemdaneprocel/MSK-DA01) enrolled 12 participants (low dose 0.9 million vs high dose 2.7 million cells per putamen) with 1 year of immunosuppression. The trial met its primary safety/tolerability endpoint, showed increased putaminal ^18^F-DOPA uptake at 18 months, a mean 23-point improvement in MDS-UPDRS Part III OFF in the high-dose cohort, and no graft-induced dyskinesias (Tabar et al., 2025). Despite shared limitations (small samples, open-label designs, and absence of placebo controls), neither study reported tumor formation or product-related serious adverse events across 18 to 24 months of follow-up. Together, these data mark key milestones and inform the next phase of development—larger randomized trials to test efficacy, durability of graft survival/integration, and to refine dosing, immunosuppression strategies, and anatomical targeting.

Even with these advances, host-to-graft spread of toxic α-syn remains a major risk for long-term efficacy of PD cell transplantation. Classic autopsy studies of fetal grafts more than 10 years post surgery revealed Lewy body–like inclusions in grafted neurons, implicating host-to-graft propagation of pathogenic α-syn (Li et al., 2008). Recent preclinical work shows that host-derived α-syn fibrils can infiltrate transplanted *SNCA-null* human mDA neurons, suggesting that simply deleting donor *SNCA* may not suffice. Although current clinical protocols have not integrated antipropagation strategies, several are under active development, including reducing extracellular seeds via antibodies/vaccines; lowering endogenous α-syn production (eg, antisense oligonucleotide such as BIIB101 (Butler et al., 2022)); blocking uptake/release pathways (eg, targeting heparan-sulfate proteoglycans, LRP1, and FAM171A2) (Tao et al., 2022, Wu et al., 2025); and improving patient selection and monitoring with biomarkers such as skin-phosphorylated α-syn and seed-amplification assay to quantify seeding burden (Gibbons et al., 2024, Parveen et al., 2025). Collectively, these developments suggest that next-generation CRT trials may need to combine cell replacement with anti–α-syn propagation strategies to achieve durable, long-term benefit.

## Ectopic vs Homotopic Grafting and Circuit Restoration

Current CRT strategies for PD predominantly employ ectopic transplantation of DA progenitors into the striatum, enabling localized, tonic dopamine release. Although this approach effectively alleviates motor symptoms, it does not restore the native architecture or dynamic regulation of the nigrostriatal pathway. In contrast, homotopic grafting into the SN aims to reconstitute physiological circuitry by allowing transplanted neurons to extend axons toward appropriate forebrain targets, thereby reconstructing the full DA circuit (Fig. 1A and D) (Gaillard et al., 2009, Thompson et al., 2009). Preclinical studies utilizing fetal VM tissue and hESC-derived mesDA neurons have demonstrated that SN grafts can re-establish a functional nigrostriatal projection, with axons reaching the striatum as well as cortical and limbic regions (Adler et al., 2020, Adler et al., 2019, Grealish et al., 2015). Importantly, grafted mesDA neurons maintain cell-type specificity, as evidenced by the failure of forebrain-derived glutamatergic neurons to innervate inappropriate regions like the caudate-putamen (Adler et al., 2019, Xiong et al., 2021). Moreover, intranigral grafts receive afferent input from cortical and striatal regions, indicating the potential to re-establish bidirectional functional connectivity. Restoration of the nigrostriatal pathway is expected to primarily improve motor function. Any effects on nonmotor symptoms are likely indirect and domain-limited—for example, improved motivation/apathy, mood, pain, or sleep continuity through stabilization of basal ganglia-thalamo-cortical dynamics and reduction of OFF-related fluctuations (eg, nocturnal akinesia)—rather than a direct correction of the widespread ND and extra-nigrostriatal pathologies that underlie most nonmotor symptoms (Leisman et al., 2014). Accordingly, core features mediated by cholinergic, serotonergic, noradrenergic, hypothalamic, or autonomic systems (eg, dementia, orthostatic hypotension) are unlikely to normalize with nigrostriatal repair alone and may require adjunctive, system-specific interventions. However, clinical translation of homotopic grafting faces significant obstacles. The human nigrostriatal pathway spans distances roughly 10 times longer than in rodent models, making long-range axonal regeneration a major challenge. To overcome this, combinatorial approaches incorporating neurotrophic factors (eg, glial cell line–derived neurotrophic factor) and axon guidance cues (eg, Netrin-1) are being explored. These adjunctive strategies have shown promise in promoting mesDA axonal outgrowth, appropriate target innervation, and synaptic integration (Ghosh et al., 2019, Jasmin et al., 2021, Thompson et al., 2009, Wang et al., 1996). Their integration may prove essential for enabling successful homotopic transplantation and full restoration of DA circuitry in human patients.

## CONCLUSION

Since the initiation of clinical trials using hPSC-derived therapies for PD, substantial technical progress has brought the field closer to clinical application. Nonetheless, achieving long-term survival and sustained function of transplanted DA neurons remains a critical challenge. Given that the underlying mechanisms of PD onset and progression remain incompletely understood in most patients, it is essential to develop strategies that mitigate pathological factors that may compromise graft viability after transplantation. Traditionally, disease-modifying strategies have focused on preserving endogenous DA neurons. Going forward, equal emphasis must be placed on protecting grafted neurons from the same hostile microenvironment—characterized by oxidative stress, α-syn aggregation, and neuroinflammation—that drives neurodegeneration. Combining cell-based therapies with established or emerging disease-modifying interventions may produce synergistic effects, enhancing graft survival, integration, and function while addressing the broader neurodegenerative cascade. Ultimately, the future of PD therapy lies in a comprehensive, multimodal approach that integrates regenerative medicine with precision neuroprotection. This includes tailoring interventions to individual genetic and molecular profiles, optimizing immunological compatibility, and promoting circuit-level reconstruction. Such a strategy offers the greatest potential to move beyond symptomatic management toward true disease modification and functional neural restoration.

## Author Contributions

**Byoung Dae Lee:** Writing – review & editing, Writing – original draft, Funding acquisition, Conceptualization. **Tae Young Kim:** Writing – original draft.

## Declaration of Competing Interests

The authors declare no competing interests.

## References

[bib1] Adler A.F., Bjorklund A., Parmar M. (2020). Transsynaptic tracing and its emerging use to assess graft-reconstructed neural circuits. Stem Cells.

[bib2] Adler A.F., Cardoso T., Nolbrant S., Mattsson B., Hoban D.B., Jarl U., Wahlestedt J.N., Grealish S., Bjorklund A., Parmar M. (2019). hESC-Derived dopaminergic transplants integrate into basal ganglia circuitry in a preclinical model of Parkinson's disease. Cell Rep..

[bib3] Aziz T.Z., Peggs D., Sambrook M.A., Crossman A.R. (1991). Lesion of the subthalamic nucleus for the alleviation of 1-methyl-4-phenyl-1,2,3,6-tetrahydropyridine (MPTP)-induced parkinsonism in the primate. Mov. Disord..

[bib4] Bari A.A., Thum J., Babayan D., Lozano A.M. (2018). Current and expected advances in deep brain stimulation for movement disorders. Prog. Neurol. Surg..

[bib5] Barker R.A., Drouin-Ouellet J., Parmar M. (2015). Cell-based therapies for Parkinson disease-past insights and future potential. Nat. Rev. Neurol..

[bib6] Benazzouz A., Gross C., Feger J., Boraud T., Bioulac B. (1993). Reversal of rigidity and improvement in motor performance by subthalamic high-frequency stimulation in MPTP-treated monkeys. Eur. J. Neurosci..

[bib7] Bergman H., Wichmann T., DeLong M.R. (1990). Reversal of experimental parkinsonism by lesions of the subthalamic nucleus. Science.

[bib8] Bjorklund A., Stenevi U. (1979). Reconstruction of the nigrostriatal dopamine pathway by intracerebral nigral transplants. Brain Res..

[bib9] Butler Y.R., Liu Y., Kumbhar R., Zhao P., Gadhave K., Wang N., Li Y., Mao X., Wang W. (2022). alpha-Synuclein fibril-specific nanobody reduces prion-like alpha-synuclein spreading in mice. Nat. Commun..

[bib10] Calabresi P., Mechelli A., Natale G., Volpicelli-Daley L., Di Lazzaro G., Ghiglieri V. (2023). Alpha-synuclein in Parkinson's disease and other synucleinopathies: from overt neurodegeneration back to early synaptic dysfunction. Cell Death Dis..

[bib11] Camilleri M., Subramanian T., Pagan F., Isaacson S., Gil R., Hauser R.A., Feldman M., Goldstein M., Kumar R., Truong D. (2022). Oral ENT-01 targets enteric neurons to treat constipation in Parkinson disease: a randomized controlled trial. Ann Intern. Med..

[bib12] Chambers S.M., Fasano C.A., Papapetrou E.P., Tomishima M., Sadelain M., Studer L. (2009). Highly efficient neural conversion of human ES and iPS cells by dual inhibition of SMAD signaling. Nat. Biotechnol..

[bib13] Chen Y., Xiong M., Dong Y., Haberman A., Cao J., Liu H., Zhou W., Zhang S.C. (2016). Chemical control of grafted human PSC-derived neurons in a mouse model of Parkinson's disease. Cell Stem Cell.

[bib14] Colucci F., Avenali M., De Micco R., Fusar Poli M., Cerri S., Stanziano M., Bacila A., Cuconato G., Franco V., Franciotta D. (2023). Ambroxol as a disease-modifying treatment to reduce the risk of cognitive impairment in GBA-associated Parkinson's disease: a multicentre, randomised, double-blind, placebo-controlled, phase II trial. The AMBITIOUS study protocol. BMJ Neurol. Open.

[bib15] Deuschl G., Schade-Brittinger C., Krack P., Volkmann J., Schafer H., Botzel K., Daniels C., Deutschlander A., Dillmann U., Eisner W. (2006). A randomized trial of deep-brain stimulation for Parkinson's disease. N. Engl. J. Med..

[bib16] Di Maio R., Hoffman E.K., Rocha E.M., Keeney M.T., Sanders L.H., De Miranda B.R., Zharikov A., Van Laar A., Stepan A.F., Lanz T.A. (2018). LRRK2 activation in idiopathic Parkinson's disease. Sci. Transl. Med..

[bib17] Doi D., Samata B., Katsukawa M., Kikuchi T., Morizane A., Ono Y., Sekiguchi K., Nakagawa M., Parmar M., Takahashi J. (2014). Isolation of human induced pluripotent stem cell-derived dopaminergic progenitors by cell sorting for successful transplantation. Stem Cell Rep..

[bib18] Eblan M.J., Walker J.M., Sidransky E. (2005). The glucocerebrosidase gene and Parkinson's disease in Ashkenazi Jews. N. Engl. J. Med..

[bib19] Gaillard A., Decressac M., Frappe I., Fernagut P.O., Prestoz L., Besnard S., Jaber M. (2009). Anatomical and functional reconstruction of the nigrostriatal pathway by intranigral transplants. Neurobiol. Dis..

[bib20] Ghosh B., Zhang C., Ziemba K.S., Fletcher A.M., Yurek D.M., Smith G.M. (2019). Partial reconstruction of the nigrostriatal circuit along a preformed molecular guidance pathway. Mol. Ther. Methods Clin. Dev..

[bib21] Gibbons C.H., Levine T., Adler C., Bellaire B., Wang N., Stohl J., Agarwal P., Aldridge G.M., Barboi A., Evidente V.G.H. (2024). Skin biopsy detection of phosphorylated alpha-synuclein in patients with synucleinopathies. JAMA.

[bib22] Grealish S., Heuer A., Cardoso T., Kirkeby A., Jonsson M., Johansson J., Bjorklund A., Jakobsson J., Parmar M. (2015). Monosynaptic tracing using modified rabies virus reveals early and extensive circuit integration of human embryonic stem cell-derived neurons. Stem Cell Rep..

[bib23] Habas A., Reddy Natala S., Bowden-Verhoek J.K., Stocking E.M., Price D.L., Wrasidlo W., Bonhaus D.W., Gill M.B. (2022). NPT1220-312, a TLR2/TLR9 small molecule antagonist, inhibits pro-inflammatory signaling, cytokine release, and NLRP3 inflammasome activation. Int. J. Inflam..

[bib24] Isaacson S.H., Jenner P. (2025). Moving to a non-dopaminergic approach for the treatment of OFF fluctuations in Parkinson's disease. Clin. Park. Relat. Disord..

[bib25] Jasmin M., Ahn E.H., Voutilainen M.H., Fombonne J., Guix C., Viljakainen T., Kang S.S., Yu L.Y., Saarma M., Mehlen P. (2021). Netrin-1 and its receptor DCC modulate survival and death of dopamine neurons and Parkinson's disease features. EMBO J..

[bib26] Jenner P. (2014). An overview of adenosine A2A receptor antagonists in Parkinson's disease. Int. Rev. Neurobiol..

[bib27] Jenner P., Rocha J.F., Ferreira J.J., Rascol O., Soares-da-Silva P. (2021). Redefining the strategy for the use of COMT inhibitors in Parkinson's disease: the role of opicapone. Expert Rev. Neurother..

[bib28] Jennings D., Huntwork-Rodriguez S., Vissers M., Daryani V.M., Diaz D., Goo M.S., Chen J.J., Maciuca R., Fraser K., Mabrouk O.S. (2023). LRRK2 inhibition by BIIB122 in healthy participants and patients with Parkinson's disease. Mov. Disord..

[bib29] Kikuchi T., Morizane A., Doi D., Magotani H., Onoe H., Hayashi T., Mizuma H., Takara S., Takahashi R., Inoue H. (2017). Human iPS cell-derived dopaminergic neurons function in a primate Parkinson's disease model. Nature.

[bib30] Kirkeby A., Grealish S., Wolf D.A., Nelander J., Wood J., Lundblad M., Lindvall O., Parmar M. (2012). Generation of regionally specified neural progenitors and functional neurons from human embryonic stem cells under defined conditions. Cell Rep.

[bib31] Kirkeby A., Nolbrant S., Tiklova K., Heuer A., Kee N., Cardoso T., Ottosson D.R., Lelos M.J., Rifes P., Dunnett S.B. (2017). Predictive markers guide differentiation to improve graft outcome in clinical translation of hESC-based therapy for Parkinson's disease. Cell Stem Cell.

[bib32] Klietz M., Greten S., Wegner F., Hoglinger G.U. (2019). Safety and tolerability of pharmacotherapies for Parkinson's disease in geriatric patients. Drugs Aging.

[bib33] Kopytova A.E., Rychkov G.N., Nikolaev M.A., Baydakova G.V., Cheblokov A.A., Senkevich K.A., Bogdanova D.A., Bolshakova O.I., Miliukhina I.V., Bezrukikh V.A. (2021). Ambroxol increases glucocerebrosidase (GCase) activity and restores GCase translocation in primary patient-derived macrophages in Gaucher disease and Parkinsonism. Parkinsonism Relat. Disord..

[bib34] Kriks S., Shim J.W., Piao J., Ganat Y.M., Wakeman D.R., Xie Z., Carrillo-Reid L., Auyeung G., Antonacci C., Buch A. (2011). Dopamine neurons derived from human ES cells efficiently engraft in animal models of Parkinson's disease. Nature.

[bib35] Leisman G., Braun-Benjamin O., Melillo R. (2014). Cognitive-motor interactions of the basal ganglia in development. Front. Syst. Neurosci..

[bib36] Levin J., Sing N., Melbourne S., Morgan A., Mariner C., Spillantini M.G., Wegrzynowicz M., Dalley J.W., Langer S., Ryazanov S. (2022). Safety, tolerability and pharmacokinetics of the oligomer modulator anle138b with exposure levels sufficient for therapeutic efficacy in a murine Parkinson model: a randomised, double-blind, placebo-controlled phase 1a trial. EBioMedicine.

[bib37] Li J.Y., Englund E., Holton J.L., Soulet D., Hagell P., Lees A.J., Lashley T., Quinn N.P., Rehncrona S., Bjorklund A. (2008). Lewy bodies in grafted neurons in subjects with Parkinson's disease suggest host-to-graft disease propagation. Nat. Med..

[bib38] Li W., Englund E., Widner H., Mattsson B., van Westen D., Latt J., Rehncrona S., Brundin P., Bjorklund A., Lindvall O. (2016). Extensive graft-derived dopaminergic innervation is maintained 24 years after transplantation in the degenerating Parkinsonian brain. Proc. Natl. Acad. Sci. U.S.A..

[bib39] Lindvall O., Brundin P., Widner H., Rehncrona S., Gustavii B., Frackowiak R., Leenders K.L., Sawle G., Rothwell J.C., Marsden C.D. (1990). Grafts of fetal dopamine neurons survive and improve motor function in Parkinson's disease. Science.

[bib40] Lindvall O., Rehncrona S., Brundin P., Gustavii B., Astedt B., Widner H., Lindholm T., Bjorklund A., Leenders K.L., Rothwell J.C. (1989). Human fetal dopamine neurons grafted into the striatum in two patients with severe Parkinson's disease. A detailed account of methodology and a 6-month follow-up. Arch. Neurol..

[bib41] Lindvall O., Widner H., Rehncrona S., Brundin P., Odin P., Gustavii B., Frackowiak R., Leenders K.L., Sawle G., Rothwell J.C. (1992). Transplantation of fetal dopamine neurons in Parkinson's disease: one-year clinical and neurophysiological observations in two patients with putaminal implants. Ann. Neurol..

[bib42] Louveau A., Smirnov I., Keyes T.J., Eccles J.D., Rouhani S.J., Peske J.D., Derecki N.C., Castle D., Mandell J.W., Lee K.S. (2015). Structural and functional features of central nervous system lymphatic vessels. Nature.

[bib43] Meissner W.G., Remy P., Giordana C., Maltete D., Derkinderen P., Houeto J.L., Anheim M., Benatru I., Boraud T., Brefel-Courbon C. (2024). Trial of lixisenatide in early Parkinson's disease. N. Engl. J. Med..

[bib44] Nikolcheva T., Pagano G., Pross N., Simuni T., Marek K., Postuma R.B., Pavese N., Stocchi F., Seppi K., Monnet A. (2025). A Phase 2b, multicenter, randomized, double-blind, placebo-controlled study to evaluate the efficacy and safety of intravenous prasinezumab in early-stage Parkinson's disease (PADOVA): rationale, design, and baseline data. Parkinsonism Relat. Disord..

[bib45] Pagano G., Taylor K.I., Anzures-Cabrera J., Marchesi M., Simuni T., Marek K., Postuma R.B., Pavese N., Stocchi F., Azulay J.P. (2022). Trial of prasinezumab in early-stage Parkinson's disease. N. Engl. J. Med..

[bib46] Pagano G., Taylor K.I., Anzures Cabrera J., Simuni T., Marek K., Postuma R.B., Pavese N., Stocchi F., Brockmann K., Svoboda H. (2024). Prasinezumab slows motor progression in rapidly progressing early-stage Parkinson's disease. Nat. Med..

[bib47] Parmar M., Grealish S., Henchcliffe C. (2020). The future of stem cell therapies for Parkinson disease. Nat. Rev. Neurosci..

[bib48] Parveen S., Alam P., Orru C.D., Vascellari S., Hughson A.G., Zou W.Q., Beach T.G., Serrano G.E., Goldstein D.S., Ghetti B. (2025). A same day alpha-synuclein RT-QuIC seed amplification assay for synucleinopathy biospecimens. NPJ Biosens..

[bib49] Perlow M.J., Freed W.J., Hoffer B.J., Seiger A., Olson L., Wyatt R.J. (1979). Brain grafts reduce motor abnormalities produced by destruction of nigrostriatal dopamine system. Science.

[bib50] Rajamani N., Friedrich H., Butenko K., Dembek T., Lange F., Navratil P., Zvarova P., Hollunder B., de Bie R.M.A., Odekerken V.J.J. (2024). Deep brain stimulation of symptom-specific networks in Parkinson's disease. Nat. Commun..

[bib51] Rampello L., Raffaele R., Furnari P., Vecchio I., Malaguarnera M. (1996). Psychotic complications of long term levodopa treatment of Parkinson's disease. Arch. Gerontol. Geriatr..

[bib52] Roy A., Dawson V.L., Dawson T.M. (2025). From metabolism to mind: the expanding role of the GLP-1 receptor in neurotherapeutics. Neurotherapeutics.

[bib53] Sawamoto N., Doi D., Nakanishi E., Sawamura M., Kikuchi T., Yamakado H., Taruno Y., Shima A., Fushimi Y., Okada T. (2025). Phase I/II trial of iPS-cell-derived dopaminergic cells for Parkinson's disease. Nature.

[bib54] Schapira A.H.V., Chaudhuri K.R., Jenner P. (2017). Non-motor features of Parkinson disease. Nat. Rev. Neurosci..

[bib55] Sidransky E., Nalls M.A., Aasly J.O., Aharon-Peretz J., Annesi G., Barbosa E.R., Bar-Shira A., Berg D., Bras J., Brice A. (2009). Multicenter analysis of glucocerebrosidase mutations in Parkinson's disease. N. Engl. J. Med..

[bib56] Smit J.W., Basile P., Prato M.K., Detalle L., Mathy F.X., Schmidt A., Lalla M., Germani M., Domange C., Biere A.L. (2022). Phase 1/1b studies of UCB0599, an oral inhibitor of alpha-synuclein misfolding, including a randomized study in Parkinson's disease. Mov. Disord..

[bib57] Tabar V., Sarva H., Lozano A.M., Fasano A., Kalia S.K., Yu K.K.H., Brennan C., Ma Y., Peng S., Eidelberg D. (2025). Phase I trial of hES cell-derived dopaminergic neurons for Parkinson's disease. Nature.

[bib58] Takahashi K., Okita K., Nakagawa M., Yamanaka S. (2007). Induction of pluripotent stem cells from fibroblast cultures. Nat. Protoc..

[bib59] Tan Y.Y., Jenner P., Chen S.D. (2022). Monoamine oxidase-B inhibitors for the treatment of Parkinson's disease: past, present, and future. J. Parkinsons Dis..

[bib60] Tao Y., Sun Y., Lv S., Xia W., Zhao K., Xu Q., Zhao Q., He L., Le W., Wang Y. (2022). Heparin induces alpha-synuclein to form new fibril polymorphs with attenuated neuropathology. Nat. Commun..

[bib61] Taymans J.M., Fell M., Greenamyre T., Hirst W.D., Mamais A., Padmanabhan S., Peter I., Rideout H., Thaler A. (2023). Perspective on the current state of the LRRK2 field. NPJ Parkinsons Dis..

[bib62] Thanvi B.R., Lo T.C. (2004). Long term motor complications of levodopa: clinical features, mechanisms, and management strategies. Postgrad. Med. J..

[bib63] Thompson L.H., Grealish S., Kirik D., Bjorklund A. (2009). Reconstruction of the nigrostriatal dopamine pathway in the adult mouse brain. Eur. J. Neurosci..

[bib64] Thomson J.A., Itskovitz-Eldor J., Shapiro S.S., Waknitz M.A., Swiergiel J.J., Marshall V.S., Jones J.M. (1998). Embryonic stem cell lines derived from human blastocysts. Science.

[bib65] Verschuur C.V., Suwijn S.R., Post B., Dijkgraaf M., Bloem B.R., van Hilten J.J., van Laar T., Tissingh G., Deuschl G., Lang A.E. (2015). Protocol of a randomised delayed-start double-blind placebo-controlled multi-centre trial for Levodopa in EArly Parkinson's disease: the LEAP-study. BMC Neurol..

[bib66] Vijiaratnam N., Girges C., Auld G., McComish R., King A., Skene S.S., Hibbert S., Wong A., Melander S., Gibson R. (2025). Exenatide once a week versus placebo as a potential disease-modifying treatment for people with Parkinson's disease in the UK: a phase 3, multicentre, double-blind, parallel-group, randomised, placebo-controlled trial. Lancet.

[bib67] Wang C.C., Wu T.L., Lin F.J., Tai C.H., Lin C.H., Wu R.M. (2022). Amantadine treatment and delayed onset of levodopa-induced dyskinesia in patients with early Parkinson's disease. Eur. J. Neurol..

[bib68] Wang Y., Tien L.T., Lapchak P.A., Hoffer B.J. (1996). GDNF triggers fiber outgrowth of fetal ventral mesencephalic grafts from nigra to striatum in 6-OHDA-lesioned rats. Cell Tissue Res..

[bib69] Wilmut I., Leslie S., Martin N.G., Peschanski M., Rao M., Trounson A., Turner D., Turner M.L., Yamanaka S., Taylor C.J. (2015). Development of a global network of induced pluripotent stem cell haplobanks. Regen. Med..

[bib70] Wu K.M., Xu Q.H., Liu Y.Q., Feng Y.W., Han S.D., Zhang Y.R., Chen S.D., Guo Y., Wu B.S., Ma L.Z. (2025). Neuronal FAM171A2 mediates alpha-synuclein fibril uptake and drives Parkinson's disease. Science.

[bib71] Xiong M., Tao Y., Gao Q., Feng B., Yan W., Zhou Y., Kotsonis T.A., Yuan T., You Z., Wu Z. (2021). Human stem cell-derived neurons repair circuits and restore neural function. Cell Stem Cell.

[bib72] Yang L., Kress B.T., Weber H.J., Thiyagarajan M., Wang B., Deane R., Benveniste H., Iliff J.J., Nedergaard M. (2013). Evaluating glymphatic pathway function utilizing clinically relevant intrathecal infusion of CSF tracer. J. Transl. Med..

[bib73] Zhang X., Wu H., Tang B., Guo J. (2024). Clinical, mechanistic, biomarker, and therapeutic advances in GBA1-associated Parkinson's disease. Transl. Neurodegener..

